# Genome-wide DNA methylation analysis reveals loci that distinguish different types of adipose tissue in obese individuals

**DOI:** 10.1186/s13148-017-0344-4

**Published:** 2017-05-03

**Authors:** Donia Macartney-Coxson, Miles C. Benton, Ray Blick, Richard S. Stubbs, Ronald D. Hagan, Michael A. Langston

**Affiliations:** 1 Biomarkers Group, Institute of Environmental Science and Research (ESR), Wellington, 5022 New Zealand; 2 Genomics Research Centre, Institute of Health and Biomedical Innovation, Queensland University of Technology, Kelvin Grove, QLD 4059 Australia; 3 The Wakefield Clinic, PO Box 7366, Wellington, 6242 New Zealand; 4 Department of Electrical Engineering & Computer Science, University of Tennessee, Knoxville, TN 37996-2250 USA

**Keywords:** Adipose, Biomarkers, DNA methylation, Graph-theoretical algorithms, Epigenetics, Obesity

## Abstract

**Background:**

Epigenetic mechanisms provide an interface between environmental factors and the genome and are known to play a role in complex diseases such as obesity. These mechanisms, including DNA methylation, influence the regulation of development, differentiation and the establishment of cellular identity. Here we employ two approaches to identify differential methylation between two white adipose tissue depots in obese individuals before and after gastric bypass and significant weight loss. We analyse genome-wide DNA methylation data using (a) traditional paired *t* tests to identify significantly differentially methylated loci (Bonferroni-adjusted *P* ≤ 1 × 10^−7^) and (b) novel combinatorial algorithms to identify loci that differentiate between tissue types.

**Results:**

Significant differential methylation was observed for 3239 and 7722 CpG sites, including 784 and 1129 extended regions, between adipose tissue types before and after significant weight loss, respectively. The vast majority of these extended differentially methylated regions (702) were consistent across both time points and enriched for genes with a role in transcriptional regulation and/or development (e.g. homeobox genes). Other differentially methylated loci were only observed at one time point and thus potentially highlight genes important to adipose tissue dysfunction observed in obesity. Strong correlations (*r* > 0.75, *P* ≤ 0.001) were observed between changes in DNA methylation (subcutaneous adipose vs omentum) and changes in clinical trait, in particular for CpG sites within *PITX2* and fasting glucose and four CpG sites within *ISL2* and HDL. A single CpG site (cg00838040, *ATP2C2*) gave strong tissue separation, with validation in independent subcutaneous (*n* = 681) and omental (*n* = 33) adipose samples.

**Conclusions:**

This is the first study to report a genome-wide DNA methylome comparison of subcutaneous abdominal and omental adipose before and after weight loss. The combinatorial approach we utilised is a powerful tool for the identification of methylation loci that strongly differentiate between these tissues. This study provides a solid basis for future research focused on the development of adipose tissue and its potential dysfunction in obesity, as well as the role DNA methylation plays in these processes.

**Electronic supplementary material:**

The online version of this article (doi:10.1186/s13148-017-0344-4) contains supplementary material, which is available to authorized users.

## Background

Epigenetic mechanisms provide an interface between environmental factors and the genome and are known to play a role in complex diseases such as obesity [1–3]. These mechanisms, including DNA methylation, influence the regulation of development, differentiation and the establishment of cellular identity (for reviews, see [4, 5]). There is increasing evidence that many complex diseases such as obesity, type 2 diabetes and coronary heart disease have at least some origins in early life—that is, the ‘developmental origin of disease’ hypothesis. This hypothesis states that such diseases result from an imbalance between the in utero and/or early life environment and that experienced later in life [6–8]. Thus, the importance of epigenetic mechanisms in development, and their significant role in complex diseases, heightens both their promise as potential diagnostic markers and their potential to provide new insights into disease biology (for a review in the context of type 2 diabetes, see [9]).

There are two main types of white adipose tissue in humans, subcutaneous and visceral (including omentum), with different depots throughout the body, for instance, abdominal and gluteal depots of subcutaneous adipose. Higher levels than expected of brown adipose tissue depots, rich in mitochondria and once thought to be found only in early development, have more recently been identified in human adults [10–12]. Furthermore, ‘browning’ or ‘beiging’ of white adipose tissue can occur [13, 14]. These various adipose tissues have distinct structural and biochemical properties [15–17], and both body fat distribution and function influence metabolic risk [18–25]. Gene expression analyses originally highlighted the potentially different developmental origins of subcutaneous and visceral adipose tissue [17, 26–28], with recent evidence suggesting a mesothelial origin for visceral adipose [29]. Furthermore, DNA methylation differences have been reported between subcutaneous abdominal and subcutaneous gluteal adipose tissue [30]. A recent study also reported genome-wide promoter methylation and transcriptome analysis of subcutaneous and omental adipose in obese vs lean individuals [31].

We previously performed DNA methylation analyses of paired subcutaneous and omental adipose from obese individuals undergoing gastric bypass, seeking to identify within-tissue differences before and after significant weight loss (>27% initial weight loss) [32]. In the current study, we perform inter-adipose tissue (subcutaneous adipose vs omentum) comparisons, hypothesising that given the different developmental origins of these tissues, (a) changes in DNA methylation before and after significant weight loss could reveal new insights into obesity biology and the role tissue development might play in this, and (b) this data would provide an excellent ‘proof of principle’ on which to test a combinatorial approach for identifying loci that differ.

## Results

### Differential methylation between subcutaneous and omental adipose at specific CpG loci

We performed paired *t* tests to identify differentially methylated CpG loci, assayed by the Illumina 450K BeadChip, between subcutaneous abdominal and omental adipose independently at two time points, before and after significant weight loss. This analysis was carried out on DNA methylation data previously obtained from 15 morbidly obese individuals before and after gastric bypass [32]. We identified 3239 (1294 annotated genes) and 7722 (3164 annotated genes) significant differentially methylated CpG loci (Bonferroni-adjusted *P* ≤ 1 × 10^−7^) between the two adipose tissue depots before and after weight loss, respectively. The majority of these CpG sites were less methylated in subcutaneous than in omental adipose, with only 23.1 and 18.8% showing relative hypermethylation in the subcutaneous depot before and after weight loss, respectively. Table 1 provides an overall summary, and Table 2 shows details on the top 20 ranking sites for each time point. The total number of CpG loci that overlapped between the analyses at the two time points was 2077 (813 annotated genes); 966 of these CpG sites showed a larger methylation difference (Δbeta) of at least 5% between the adipose tissue depots after weight loss than before. All sites passing the Bonferroni adjustment threshold are available in Additional files 1 and 2 (sites which overlap between the two time points are indicated).

**Table 1:** Summary of significantly differentially methylated CpG sites identified in subcutaneous vs omental adipose tissue before and after weight loss

	Total CpG sites	CpG sites with Δ beta ≥ 5%	CpG sites with Δ beta ≥ 10%	CpG sites with Δ beta ≥ 20%	CpG sites with Δ beta ≥ 50%
Before weight loss	3239				
+ Δbeta	2491	2400	1667	260	1
− Δbeta	748	748	682	275	0
After weight loss	7722				
+ Δbeta	6274	6193	4774	1137	7
− Δbeta	1448	1446	1340	666	3

**Table 2:** Top 20 CpG sites by magnitude of DNA methylation difference between subcutaneous and omental adipose tissue at each time point

Illumina probe ID	*T* statistic	Adjusted *P* value	AB mean beta	OM mean beta	Δbeta	CpG site chromosome and position^a^	UCSC gene name^a^ ^b^	Location	Rank *P* value^c^	Rank biomarker^d^
Before weight loss										
cg02245004	−14.92	2.32E−04	0.12	0.64	−0.51	15:76634887	Intergenic		318	1
cg20540209	−13.61	7.74E−04	0.06	0.53	−0.48	15:76633981	ISL2	Body	557	NA
cg17446010	−16.17	7.97E−05	0.13	0.58	−0.45	15:76633627	ISL2	Body	192	NA
cg00838040	−18.85	1.02E−05	0.53	0.96	−0.43	16:84446919	ATP2C2	Body	66	NA
cg03923561	23.49	5.10E−07	0.45	0.04	0.41	12:54447220	HOXC4	5′UTR;TSS1500	12	2
cg09400037	−17.63	2.50E−05	0.42	0.82	−0.40	16:84822801	Intergenic		102	4
cg22747076	20.83	2.62E−06	0.42	0.02	0.40	12:54447873	HOXC4	1stExon;Body	31	3
cg15233062	22.19	1.11E−06	0.48	0.09	0.39	12:54447349	HOXC4	5′UTR;TSS1500	18	NA
cg00108351	−16.19	7.79E−05	0.23	0.61	−0.39	16:86529658	Intergenic		191	NA
cg01184975	23.53	4.97E−07	0.44	0.06	0.39	12:114852091	Intergenic		11	NA
cg12984729	−13.17	1.19E−03	0.12	0.50	−0.38	15:76633817	ISL2	Body	668	13
cg08307030	−10.46	2.27E−02	0.14	0.52	−0.38	15:76634380	ISL2	3′UTR	2336	16
cg20291855	−20.02	4.48E−06	0.02	0.40	−0.38	4:13524143	Intergenic		41	NA
cg11797364	−14.85	2.46E-04	0.59	0.97	−0.38	6:436969	Intergenic		329	6
cg01697719	12.27	2.97E-03	0.38	0.01	0.37	22:19754125	TBX1	Body	986	NA
cg07266404	28.62	3.38E−08	0.43	0.07	0.37	12:54447584	HOXC4	5′UTR;TSS200	4	NA
cg24864887	−13.27	1.07E−03	0.14	0.50	−0.36	15:76635638	Intergenic		644	NA
**cg21215550**	18.67	1.15E−05	0.55	0.20	0.36	9:969544	Intergenic		70	NA
cg06800235	16.38	6.67E−05	0.56	0.20	0.35	1:7692367	CAMTA1	Body	176	NA
cg08036309	−21.31	1.92E−06	0.17	0.52	−0.35	4:13536991	Intergenic		24	NA
After weight loss										
cg00838040	−38.29	6.04E−10	0.34	0.97	−0.64	16:84446919	ATP2C2	Body	2	1
cg09400037	−34.83	2.24E−09	0.33	0.92	−0.59	16:84822801	Intergenic		3	NA
cg02245004	−11.25	9.01E−03	0.07	0.65	−0.58	15:76634887	Intergenic		4290	NA
cg20540209	−10.86	1.41E−02	0.02	0.59	−0.56	15:76633981	ISL2	Body	4998	NA
cg22747076	17.06	3.89E−05	0.59	0.06	0.53	12:54447873	HOXC4	1stExon;Body	517	NA
**cg24145118**	11.73	5.30E−03	0.84	0.31	0.52	10:2777041	Intergenic		3554	NA
cg17446010	−12.37	2.68E−03	0.09	0.61	−0.52	15:76633627	ISL2	Body	2760	NA
cg03923561	14.78	2.62E−04	0.60	0.09	0.51	12:54447220	HOXC4	5′UTR;TSS1500	1131	NA
cg21917524	−28.43	3.70E−08	0.31	0.81	−0.51	11:74200334	Intergenic		19	2
cg00108351	−11.97	4.11E−03	0.17	0.67	−0.51	16:86529658	Intergenic		3233	NA
cg12984729	−11.88	4.50E−03	0.09	0.58	−0.49	15:76633817	ISL2	Body	3338	4
cg15233062	14.15	4.63E−04	0.62	0.14	0.49	12:54447349	HOXC4	5′UTR;TSS1500	1400	NA
cg00319661	17.95	1.96E−05	0.92	0.43	0.49	5:2632178	Intergenic		381	NA
cg11797364	−21.71	1.49E−06	0.47	0.93	−0.47	6:436969	Intergenic		122	NA
cg13593391	12.35	2.74E−03	0.94	0.47	0.47	11:132582488	OPCML	Body	2783	NA
cg08307030	−10.93	1.30E−02	0.14	0.60	−0.46	15:76634380	ISL2	3′UTR	4855	NA
**cg08773462**	−11.52	6.65E−03	0.10	0.56	−0.46	16:17107610	Intergenic		3864	NA
cg01697719	16.48	6.15E−05	0.49	0.03	0.45	22:19754125	TBX1	Body	622	NA
cg20249252	15.65	1.22E−04	0.60	0.15	0.45	12:114852308	Intergenic		837	NA
cg17185060	−17.72	2.34E−05	0.27	0.72	−0.45	3:193990712	Intergenic		415	NA

Illumina probe IDs in bold indicate probes unique to the analysis at that time point

^a^Gene symbol and map position as Illumina manifest, GRCh37/hg19

^b^‘Intergenic’, no gene loci annotation in Illumina manifest

^c^ *P* value in ‘traditional’ differential methylation analysis

^d^Indicates ranking in combinatorial analysis if applicable. NA indicates that the CpG site was not seen in this analysis

Given the suggested mesothelial origin of visceral adipose, we interrogated the list of significant differentially methylated loci for CpG sites located within genes related to this. In particular, we looked at *WT1* (Wilms tumour 1), whose role in the development and mesothelial origin of visceral adipose was recently recognised [29, 33]. We also observed differential methylation in two mesothelial markers, *MSLN* (the gene encoding mesothelin) and *UPK3b* [34], as well as *MSLNL* (mesothelin-like). Differential methylation within *MIER2* (mesoderm induction early response 1, family member 2) was also seen in the after weight loss analysis (Table 3).

**Table 3:** Significant differential methylation within genes involved in mesothelial lineage and development

				Before weight loss				After weight loss			
Differential analysis											
Illumina probe ID	CpG site chromosome and position^a^	UCSC gene name	Location	AB mean beta	OM mean beta	Δbeta	Adjusted *P* value	AB mean beta	OM mean beta	Δbeta	Adjusted *P* value
cg01168289	11:32421514	*WT1*	Body					0.584	0.745	−0.161	0.030
cg27019929	11:3421518	*WT1*	Body					0.626	0.845	−0.221	2.1 × 10^−4^
**cg24325551**	11:3421635	*WT1*	Body	0.502	0.713	−0.211	0.002	0.425	0.665	−0.241	2.1 × 10^−5^
cg20449659	11:3421752	*WT1*	Body	0.594	0.795	−0.201	0.03	0.469	0.751	−0.282	5.7 × 10^−6^
**cg08575722**	11:3421845	*WT1*	Body					0.36	0.622	−0.262	0.003
cg09695430	11:32447944	*WT1*	Body	0.326	0.527	−0.2	1.2 × 10^−4^	0.259	0.557	−0.298	1.2 × 10^−5^
**cg2020498**6	11:32448067	*WT1*	Body	0.406	0.596	−0.19	0.001	0.314	0.596	−0.281	2.1 × 10^−4^
**cg13638420**	11:32448293	*WT1*	Body	0.236	0.391	−0.155	0.014	0.195	0.447	−0.253	0.009
**cg12006284**	11:32449638	*WT1*	Body	0.303	0.448	−0.145	0.022				
**cg13540960**	11:32450244	*WT1*	Body	0.082	0.270	−0.188	0.05				
**cg05222924**	11:32450486	*WT1*	Body	0.072	0.238	−0.166	1.3 × 10^−4^				
cg19211915	11:32452513	*WT1*	Body	0.183	0.250	−0.067	0.03				
**cg0923461**6	11:32452592	*WT1*	Body	0.074	0.203	−0.129	0.025				
cg08079266	16:817098	*MSLN*	Body	0.877	0.922	−0.045	0.03				
**cg04476874**	19:345778	*MIER2*	TSS1500					0.527	0.735	−0.208	2.7 × 10^−5^
Biomarker approach											
Illumina probe ID	CpG site chromosome and position^a^	UCSC gene name	Location	AB mean beta	OM mean beta	Δbeta	Merit score	AB mean beta	OM mean beta	Δbeta	Merit score
cg05069835	7:76139338	*UPK3B*	TSS1500					0.49	0.557	−0.067	0.006
**cg24325551**	11:3421635	*WT1*	Body	0.502	0.713	−0.211	0.109				
cg10244666	11:3421808	*WT1*	Body					0.639	0.357	0.282	0.130
**cg08575722**	11:3421845	*WT1*	Body	0.479	0.648	−0.169	0.035				
**cg20204986**	11:32448067	*WT1*	Body	0.406	0.596	−0.19	0.086				
**cg13638420**	11:32448293	*WT1*	Body					0.447	0.195	0.252	0.147
**cg12006284**	11:32449638	*WT1*	Body					0.506	0.274	0.232	0.074
**cg04456238**	11:32450104	*WT1*		0.301	0.437	−0.136	0.025	0.481	0.251	0.23	0.069
cg13540960	11:32450244	*WT1*		0.082	0.27	−0.188	0.099				
**cg05222924**	11:32450486	*WT1*						0.316	0.091	0.225	0.099
cg06516124	11:32450591	*WT1*		0.089	0.184	−0.095	0.017				
**cg09234616**	11:32452592	*WT1*		0.074	0.203	−0.129	0.049				
cg15315385	11:32452839	*WT1*						0.27	0.169	0.101	0.016
cg04130356	16:810643	*MSLN*	TSS1500					0.686	0.789	−0.103	0.04
cp0838005	16:816149	*MSLN*	Body					0.946	0.883	0.063	0.006
cg08581018	16:832942	*MSLNL*	TSS200					0.621	0.722	−0.101	0.015
cg16804923	16:834399	*MSLNL*	TSS1500	0.838	0.898	−0.06	0.012	0.861	0.756	0.105	0.029
**cg04476874**	19:345778	*MIER2*	TSS1500					0.735	0.527	0.208	0.134

Illumina probe IDs in bold indicate probes which overlap between the two analytical approaches

^a^Gene symbol and map position as Illumina manifest, GRCh37/hg19

### Extended regions of differential methylation between subcutaneous and omental adipose

**Fig. 1:**
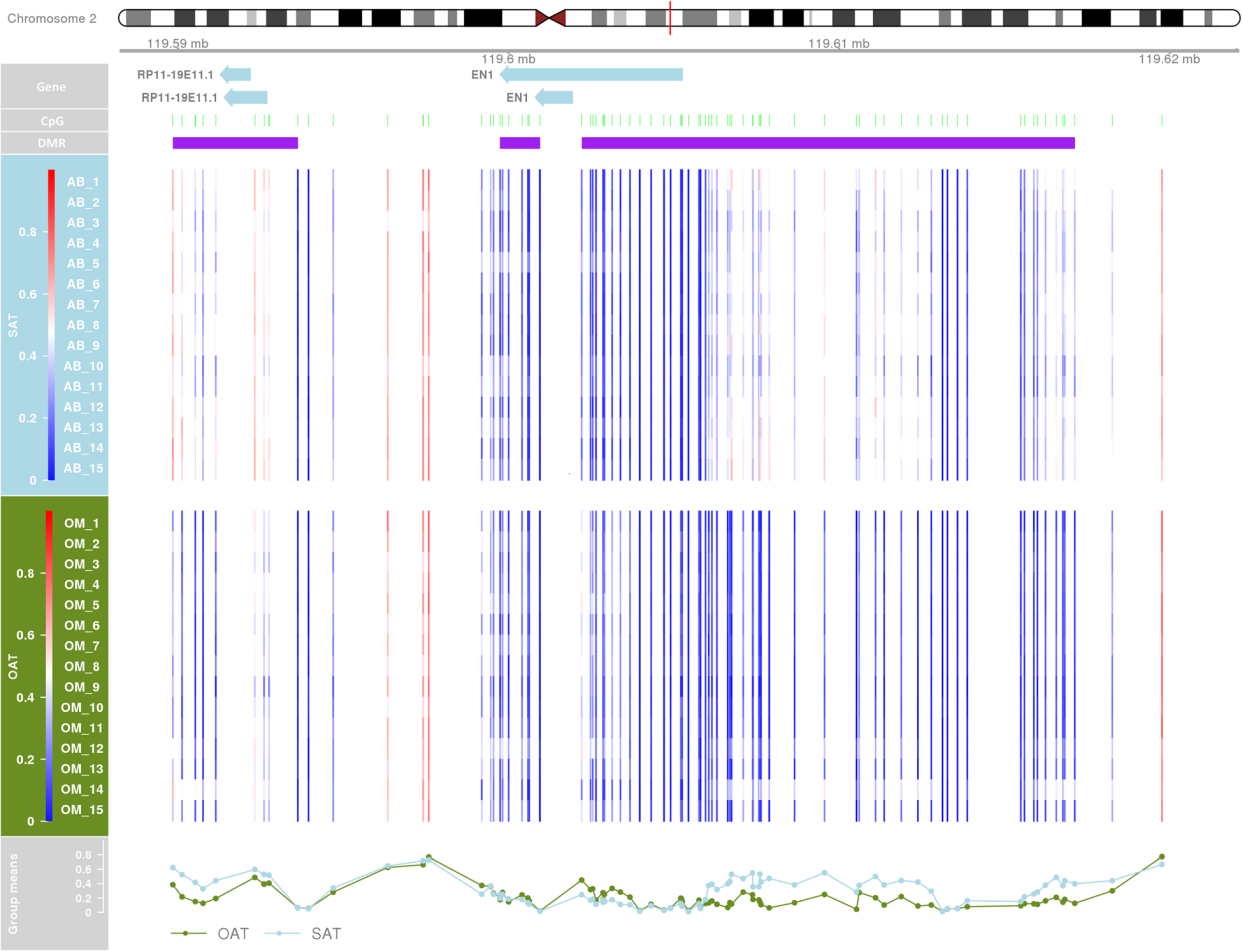
Plot illustrating a DMR located in *EN1*. Samples from the after gastric bypass and weight loss analyses (*OP2*) are shown. The chromosomal and gene architecture is shown with relative methylation illustrated below for each subcutaneous adipose (*AB*) and omental (*OM*) sample from hyper- (*red*) through hypomethylation (*blue*). Average methylation (mean beta value) is plotted for each tissue, omentum (*OAT*, *green*) and subcutaneous adipose (*SAT*, *blue*), at the *bottom*

To investigate further the DNA methylome differences between subcutaneous and omental adipose, we looked for extended differentially methylated regions (DMRs) using the DMRcate package [35] in R. A total of 784 and 1126 DMRs were identified before and after weight loss, respectively, that passed a combined adjusted *P* (Stoeffler) threshold of 0.05. Of these DMRs, 706 overlapped between the comparisons. Notably, the top 3 ranking DMRs in terms of significance (Stoeffler *P* value) were located on chromosome 2 within regions to which homeobox genes mapped: *EN1* (DMR: Chr2:119602212-119617128, Fig. 1), *HOXD3* (DMR: Chr2:177021702-177030228) and *HOXD4*:*miR10b* (Chr2:177012117-177017797). The fourth was located in homeobox gene *PITX2* on chromosome 4 (Chr4:111549880-111555503). A DMR was also observed at both time points in *MSLN* (Chr16:809476-811744, before weight loss mean Δbeta = 0.059, after weight loss mean Δbeta = 0.076). Details of all significant DMRs identified are in Additional files 3 and 4.

To explore the potential biological significance of these observations, we performed enrichment analyses for the genes mapping to significant DMRs, setting Bonferroni *P* ≤ 0.05 for significant enrichment. Three gene sets were submitted for analyses to facilitate an exploration of biological functions: (i) those potentially ‘hard-wired’ to the tissue types, that is, genes mapping to significant DMRs identified in both comparisons, *n* = 385; (ii) those unique to the tissue types before gastric bypass and weight loss, that is, genes mapping to unique DMRs, *n* = 30; and (iii) those unique to the tissue types after gastric bypass and weight loss, that is, genes mapping to unique DMRs, *n* = 256. A full list of significant pathways identified for all three can be found in Additional files 5, 6 and 7.

#### Significant DMRs observed between tissues at both time points

Enrichment was observed for GO Molecular Function pathways related to DNA binding, transcription and/or transcriptional regulation, for GO Biological Processes involved in development/morphogenesis and for homeodomain/homeobox domain proteins and *PAX* genes. Gene targets for miR374 and miR10 were also enriched.

We also performed pathway analysis on the 85 overlapping DMRs (mapping to annotated genes) which showed a greater difference in the mean Δmethylation between the two tissues after weight loss compared to before. As above, enrichment was observed for pathways related to DNA binding, transcription and/or transcriptional regulation, and involved in development/morphogenesis as well as for homeodomain/homeobox domain proteins. Domain analysis also revealed enrichment for *Sim1* and *Sim2*, and *TBX* genes (*TBX15*, *TBX4*, *TBX5*). See Additional file 8 for a full list of significant pathways identified.

#### Significant DMRs unique to the subcutaneous vs omental adipose comparison before weight loss

Enrichment was observed for GO Biological Processes related to dendrite function, specifically three genes (*SYT4*, *CPNE6*, *NEDD4L*) and GO Cellular Component pathways related to the synapse. *DGALP2* was significant for protein interactions, and enrichment of gene targets for miR141 and miR200a was observed. Loci in which significant DMRs were only observed for the before weight loss comparison included *HOXD8*, *HOTAIRM1* and *HSD17B1*.

#### Significant DMRs unique to the subcutaneous vs omental adipose comparison after weight loss

Enrichment was observed for GO Molecular Function pathways involved in DNA binding, notably the retinoic acid receptor response element (GO: 0044323) and GO Biological Processes including cell adhesion, development/morphogenesis, and immune cell activity and differentiation (notably T cell function). No enrichment for protein interactions or miRNA targets was seen. Genes in which significant DMRs were only observed for the after weight loss comparison included *HOXB5*, *TBX5*, *MTOR*, *RPTOR* and *HSD17B14*.

### Identification of correlations between differential tissue methylation and clinical parameters

We reasoned that significant differentially methylated sites which overlapped between the two time points might be indicative of ‘hard-wiring’ in terms of development/differentiation of the adipose depots and that those with a greater difference at one time point might provide insight into disease pathology. Concentrating on those sites (*n* = 966) which showed a greater methylation difference after weight loss, we performed Pearson’s correlation analyses between the Δbeta (subcutaneous adipose vs omentum) and Δclinical trait (clinical trait before vs after weight loss). Filtering for *P* ≤ 0.001, before weight loss, we observed seven associations. Δbeta at one site (cg21475076) was correlated with ΔBMI and Δkg (*r* = 0.84, *r* = 0.86, respectively) with Δbeta at other sites correlated with changes in fasting insulin (cg14954582, *r* = −0.90; cg04757806, *r* = 0.83), LDL (cg1384319, *r* = −0.80) and cholesterol (cg19640526, *r* = −0.79; cg01020413, *r* = 0.78). Only one of the sites, cg04757806, mapped to an annotated gene, *FUT4*.

After weight loss, we observed 75 correlations passing a *P* ≤ 0.001 threshold. Notably, the majority of these (64/75) were with ΔHDL, including strong correlations between Δbeta and ΔHDL for four sites within 1061 bp of each other in *ISL2* (cg06375967, cg17446010, cg12984729, cg08307030 with *r* = −0.78, −0.83, −0.87, −0.86, respectively). Δbeta at two CpG sites in *PITX2* was correlated with Δfasting glucose (cg268311119 and cg24005685, *r* = −0.89 and −0.79, respectively), and one of these was also correlated with Δfasting insulin (cg24005685, *r* = −0.89). Details of all sites passing filtering at *P* ≤ 0.001 can be found in Additional file 9.

### Identification of tissue differentiation markers

**Fig. 2:**
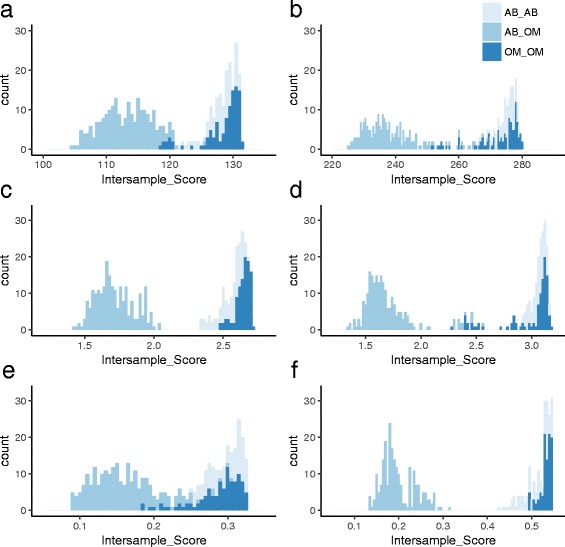
Histograms presenting the inter-sample scores for all sites passing initial analysis (**a**, **b**), the top 10 ranking sites (**c**, **d**) and the top ranking site (**e**, **f**), before (**a**, **c**, **e**) and after (**b**, **d**, **f**). Inter-sample scores were generated for all sample pairwise comparisons for omentum (*OM*) and subcutaneous abdominal adipose (*AB*). Same tissue comparisons (i.e. OM vs OM and AB vs AB) would need to score more highly than contra-tissue comparisons (OM vs AB) for a useful discriminatory panel of markers. An ideal panel would show no overlap between same and contra-group comparisons

We also analysed the DNA methylation data using a combinatorial approach, in an effort to complement and extend more standard statistical differential (paired *t* test) techniques. Having originally applied this approach to gene expression data [36], here we aimed to identify signature(s) predictive of tissue type at the two time points (before and after gastric bypass-associated weight loss). The subcutaneous abdominal vs omental adipose tissue comparisons were applied independently (before and after gastric bypass-associated weight loss). Initial analyses of the different adipose tissues before weight loss revealed 4900 CpG sites (mapping to 2309 genes) that passed filtering and had positive merit scores (such a score estimates a site’s relative ability to distinguish among sample tissue classes) (Additional file 10). Figure 2a presents the distribution of inter-sample paired comparison scores using this panel of 4900 CpG sites, which demonstrates their ability to give appealing, but not complete, separation of the tissues. An equivalent analysis of the tissues after weight loss identified 8624 CpG sites (mapping to 4066 genes) with positive merit scores (Additional file 11) that again gave an attractive separation of tissues (Fig. 2b). The total number of CpG sites that overlapped between the two analyses was 1022. Details of the top 10 ranking sites for each analysis are presented in Table 4.

**Table 4:** Top10 CpG sites by biomarker ranking that differentiate subcutaneous and omental adipose tissue at each time point

Illumina probe ID	AB mean beta (±standard deviation)	OM mean beta (±standard deviation)	UCSC gene name^a^ ^b^	CpG site chromosome and position^b^	Traditional analysis rank by *P* value	Traditional analysis rank by absolute Δbeta
Before weight loss analysis						
cg02245004	0.12 (±0.08)	0.64 (±0.11)	Intergenic	15:76634887	318	1
cg03923561	0.45 (±0.07)	0.04 (±0.02)	*HOXC4*	12:54447220	12	5
cg22747076	0.42 (±0.09	0.02 (±0.02)	*HOXC4*	12:54447873	31	7
cg09400037	0.42 (±0.06)	0.82 (±0.07)	Intergenic	16:84822801	102	6
cg24376776	0.03 (±0.01)	0.33 (±0.03)	Intergenic	10:101297245	1	62
cg11797364	0.59 (±0.1)	0.97 (±0.02)	Intergenic	6:436969	329	14
cg17496661	0.65 (±0.08)	0.99 (±0.01)	Intergenic	5:3326343	146	28
cg09720701	0.37 (±0.05)	0.06 (±0.01)	*HOXC4*	12:54447283	7	47
cg02264990	0.35 (±0.06)	0.03 (±0.02)	*HOXC4*	12:54447243	30	46
cg01524853	0.43 (±0.05)	0.12 (±0.03)	*HOXC4*	12:54447807	2	51
After weight loss analysis						
cg00838040	0.34 (±0.07)	0.97 (±0.03)	*ATP2C2*	16:84446919	2	1
cg21917524	0.31 (±0.07)	0.81 (±0.04)	Intergenic	11:74200334	19	10
cg24145118	0.84 (±0.07)	0.31 (±0.16)	Intergenic	10:2777041	3554	7
cg12984729	0.09 (±0.03)	0.59 (±0.16)	*ISL2*	15:76633817	3338	11
cg12437821	0.46 (±0.04)	0.05 (±0.09)	Intergenic	12:114852027	185	48
cg01184975	0.53 (±0.05)	0.10 (±0.11)	Intergenic	12:114852091	567	33
cg20291855	0.03 (±0.03)	0.44 (±0.11)	Intergenic	4:13524143	1994	42
cg25365014	0.79 (±0.05)	0.41 (±0.05)	Intergenic	5:2727713	1	89
cg16297011	0.07 (±0.04)	0.48 (±0.11)	Intergenic	4:13539023	1771	40
cg21982455	0.56 (±0.04)	0.19 (±0.07)	Intergenic	5:2757976	92	86

^a^‘Intergenic’, no gene loci annotation in Illumina manifest

^b^Gene symbol and map position as Illumina manifest, GRCh37/hg19

Initial analysis identified all sites with positive merit scores and then selected sites that best covered others. Given that an ideal, and potentially cost-effective, biomarker panel includes the least number of markers required for robust differentiation between groups, we investigated the discriminatory power of a reduced number of sites for each time point. A robust differentiation between tissues was identified for the top 10 ranking sites from the before weight loss analysis (Fig. 2c). Separation of the two tissues using the top 10 ranking sites from the after weight loss analysis provided better discrimination than the 8624 sites identified in the first-pass, but not complete, partitioning (Fig. 2d).

We then reduced the number of sites to one; the top site from the before weight loss comparison did not perform as well (Fig. 2e), whereas full discriminatory power was seen for the top site from the after weight loss analysis (Fig. 2f).

Given the striking utility of a single site (cg00838040) to discriminate fully the two tissue types after weight loss, we investigated this further. This CpG loci is within an intron of *ATP2C2*. Visualisation of the region surrounding the CpG in the UCSC Genome Browser (http://genome.ucsc.edu/) revealed that it sits within a region protected by transcription factor CEBPB (Chr16:84446744-84447019, CpG at Chr16:84446919, genome build 37/hg19) as indicated by ENCODE ChipSeq analyses.

Given the paired nature of the samples (i.e. subcutaneous and omental adipose from the same individuals) in the analysis, we retained probes annotated to contain SNPs (as per Illumina manifest). Therefore, we then investigated whether any SNPs were adjacent to cg00838040 in *ATP2C2*. SNP rs12102757 (C/T) is located 35 bases from the CpG site interrogated by cg00838040 and 3 bases downstream of the CEBPB binding motif. This SNP has a mean minor allele frequency of 0.282, with a range 0.168–0.511 depending on the population examined (www.ensembl.org, 1000 genomes phase III). No prior associations for rs12102757 were observed with obesity or related phenotypes when searching the latest build of the GWAS (genome-wide association study) catalog (https://www.ebi.ac.uk/gwas). We also looked at *ATP2C2* RNA abundance on the GTEx Portal (Genotype-Tissue Expression project, www.gtexportal.org) and observed minimal expression in subcutaneous adipose and omentum. When we investigated the possibility of relationships between Δbeta cg00838040 and changes in clinical traits using a Pearson’s correlation analysis, we did not observe any. Our previous study which investigated intra-tissue methylation differences before and after weight loss did not observe any correlations with clinical parameters either [32].

As for differential methylation, we examined the lists of 2309 and 4066 genes, respectively, in the before and after weight loss analyses to see whether they contained loci with a reported role in the mesothelial origin of visceral adipose tissue. We observed differential methylation within *WT1* at both time points and within *MSLN*, *MSLN1*, *UPK3B* and *MIER2* after weight loss; CpG sites in both *MSLN1 and MIER2* were also identified in the before weight loss analysis (see Table 3).

### Validation of tissue differentiation markers

We performed a technical validation of our observations using pyrosequencing of robust tissue discriminators from each analysis, that is, the top 10 ranking sites from the before weight loss analysis and the top site from the after weight loss analysis (Table 4, Figs. 2c and 1e). This revealed an excellent agreement between the two methylation assays: before weight loss (*R* ^2^ = 0.91–0.98, *P* = 1.2 × 10^−15^–8.3 × 10^−25^) and the single site after weight loss (*R* ^2^ = 0.97, *P* = 4.9 × 10^−14^).

**Fig. 3:**
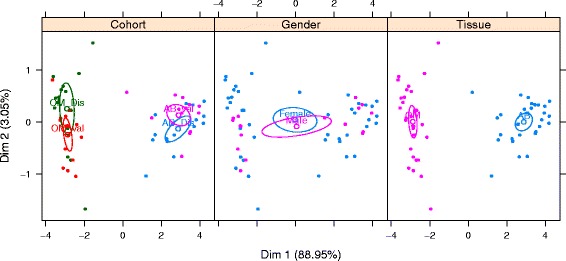
PCA of the top 10 CpG sites from the before weight loss analysis. Analysis was performed on pyrosequence data. Tissue type is indicated by OM (omentum) and *AB* (subcutaneous abdominal adipose), with *Dis* indicating the discovery and *val* the validation samples

**Fig. 4:**
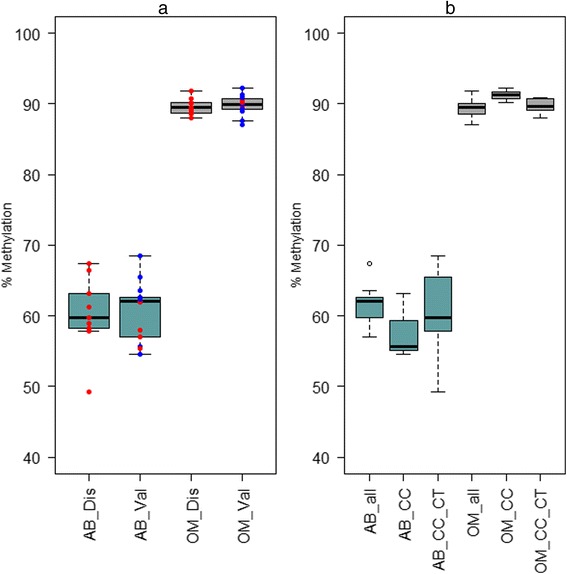
Pyrosequence data for cg00838040 in **a** the discovery and validation samples and **b** the rs1202757 genotype. Percent methylation values (*Y axis*) obtained from pyrosequence data are presented for subcutaneous adipose (*AB*, *turquoise*) and omentum (*grey*, *OM*). A. Methylation values corresponding to male and female samples are coloured *blue* and *red*, respectively. *Dis* indicates discovery and *val*, validation samples. **b** Samples are grouped by tissue and the potential genotype for rs12102757, which is located 35 bases from cg00838040

**Fig. 5:**
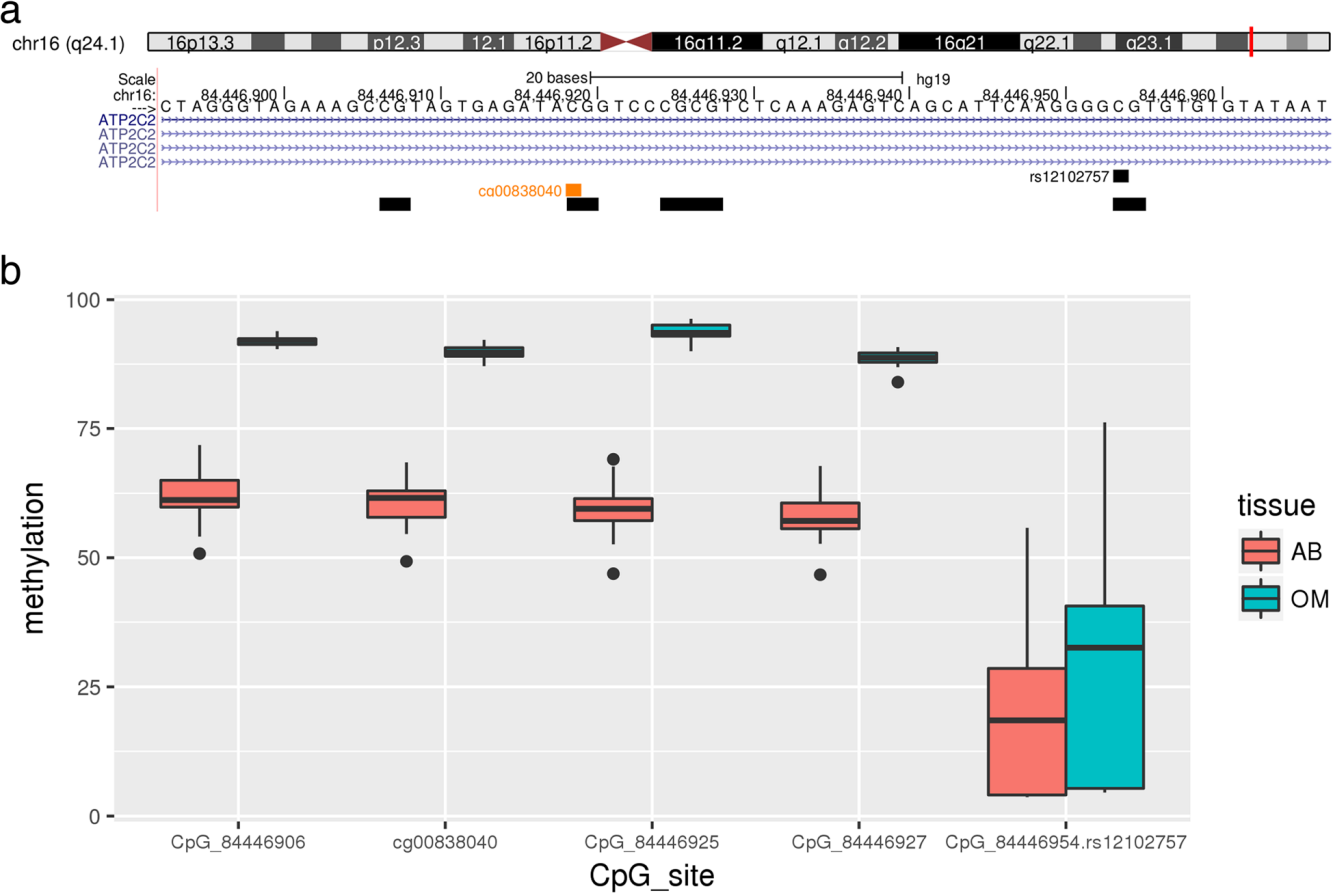
Summary of methylation across the genomic region of *ATP2C2* interrogated by pyrosequencing. **a** UCSC genome browser track region. *Yellow blocks* indicate CpG sites interrogated, *black box* indicates the location of SNP rs12102757 and *orange box* indicates the position of cg00838040. **b** Pyrosequence methylation data for the five CpG sites sequenced in the region. *AB* abdominal adipose, represented by *pink boxes*, *OM* omental adipose, represented by *blue boxes*

Next, we sought to test the performance of these DNA methylation biomarkers in additional, independent samples. DNA was available for a further 15 individuals before weight loss and 13 of these 15 individuals after weight loss. Table 5 provides a summary of the clinical and anthropometric data for these. Principal component analysis (PCA) revealed a strong agreement between the discovery and validation samples for the before weight loss comparison, with no significant variation observed between genders in the validation samples (Fig. 3). A comparison of the profile of the single site (cg00838040) in the after weight loss samples also revealed excellent agreement between the discovery and validation samples (Fig. 4a). Given that a SNP (rs1210757, mean minor allele frequency 0.282) is located within 35 bases of cg00838040, we looked at the pyrosequence data for this SNP. The C/T polymorphism forms part of a CpG dinucleotide, and, given the bisulphite conversion of the DNA prior to pyrosequencing (because of conversion of unmethylated cytosine to uracil, a T in the sequence is inconclusive in terms of genotype), it was therefore impossible to distinguish fully any genotype except CC (where full methylation is observed). Despite this, the pyrosequence data for the paired subcutaneous and omental adipose samples clearly indicates that tissue methylation at cg00838040 dominates over the rs12102757 genotype (Fig. 4b). This is further demonstrated in Fig. 5, which presents a more detailed summary of the pyrosequence data for CpG methylation across the region including cg00838040 and rs12102757. Robust tissue differentiation is observed for three CpG sites flanking cg00838040, and disruption of methylation at rs12102757, presumably via the polymorphism, is observed.

**Table 5:** Clinical and anthropometric data for individuals in the study

Trait	Before weight loss	After weight loss	*P* value (paired *t* test)
Discovery cohort, *n* = 15			
Gender	Female (*n* = 15), male (*n* = 0)		
Age (years)	44 (±10)	45 (±10)	
Weight (kg)	123.9 (±33.32)	76.2 (±15.4)	6.9 × 10^−9^
BMI (kg/m^2^)	47.6 (±11.3)	29.3 (±5.5)	3.0 × 10^−7^
Glucose (mmol/L)	5.5 (±1.2)	4.6 (±0.6)	0.035
Insulin (pmol/L)	176.2 (±168.1)	33.4 (±19.3)	0.011
HbA1c % (DCCT)^a^	6.0 (±0.6)	5.5 (±0.5)	0.036
Triglycerides (mmol/L)	1.6 (±0.7)	1.0 (±0.3)	0.01
Total cholesterol (mmol/L)	5.3 (±0.8)	4.6 (±0.6)	9.0 × 10^−4^
HDL (mmol/L)	1.4 (±0.4)	1.8 (±±0.9)	0.167
LDL (mmol/L)	3.2 (±1.1)	2.6 (±0.6)	0.018
Systolic blood pressure (mmHg)	135 (±18)	115 (±9)	0.006
Diastolic blood pressure (mmHg)	78 (±13)	73 (±9)	0.122
Time (months)		17.6 (±6.9)	
In-house validation cohort, *n* = 15^b^			
Gender	Female (*n* = 6), male (*n* = 9)		
Age (years)	Female 45 (±6)	Female 47 (±6)	
	Male 47 (±10)	Male 49 (±10)	
Weight (kg)	Female 118.7 (±13.9)	Female 77.5 (±9.7)	1.9 × 10^−4^
	Male 156.2 (±17.3)	Male 95.8 (±12.4)	3.7 × 10^−5^
BMI (kg/m^2^)	Female 43.8 (±4.0)	Female 28.9 (±2.9)	2.1 × 10^−4^
	Male 50.3 (±7.6)	Male 30.7 (±2.6)	1.0 × 10^−4^
Glucose (mmol/L)	Female 5.8 (±1.8)	Female 4.7 (±0.4)	0.169
	Male 5.8 (±0.9)	Male 5.0 (±0.5)	0.038
Insulin (pmol/L)	Female 69.7 (±39.7)	Female 25.2 (±17.1)	0.017
	Male 184.0 (±72.0)	Male 49.5 (±29.6)	0.013
HbA1c % (DCCT)^a^	Female 6.4 (±1.6)	Female 5.5 (±0.6)	0.148
	Male 6.2 (±0.5)	Male 5.5 (±0.4)	0.163
Triglycerides (mmol/L)	Female 1.3 (±1.0)	Female 0.8 (±0.3)	0.121
	Male 1.7 (±1.0)	Male 1.2 (±0.3)	0.039
Total cholesterol (mmol/L)	Female 4.6 (±1.0)	Female 4.9 (±1.5)	0.677
	Male 5.0 (±1.1)	Male 4.3 (±4.3)	0.092
HDL (mmol/L)	Female 1.4 (±0.2)	Female 1.5 (±0.2)	0.042
	Male 1.1 (±0.1)	Male 1.5 (±0.4)	0.038
LDL (mmol/L)	Female 2.6 (±0.8)	Female 3.0 (±1.4)	0.548
	Male 3.1 (±0.9)	Male 2.3 (±0.5)	0.055
Systolic blood pressure (mmHg)	Female 123 (±11)	Female 112 (±13)	
	Male 127 (±24)	Male 121 (±14)	
Diastolic blood pressure (mmHg)	Female 76 (±6)	Female 69 (±11)	
	Male 77 (±6)	Male 74 (±12)	
Time (months)		Female 19.8 (±5.5)	
		Male 17.4 (±6)	

Data are expressed as means ± standard deviation. Paired adipose samples were available for all 15 before weight loss and for 13/15 after weight loss

^a^HbA1c is reported as % using DCCT (Diabetes Control and Complications Trial) units

^b^Blood pressure data was not available for samples in the validation cohort

**Fig. 6:**
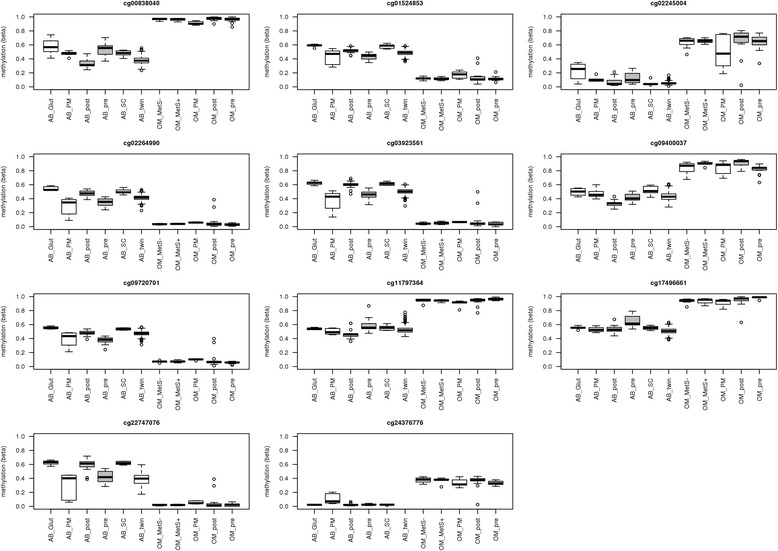
Methylation of the robust discriminatory probes from each analysis in publically available adipose tissue data. Box plots of the methylation beta values (Illumina 450K analysis) for cg00838040 and the top 10 probes from the before weight loss analysis. Subcutaneous adipose is prefaced AB in all cases and omental or visceral adipose OM. Data from the current study is shaded *grey* with before weight loss samples indicated by ‘pre’ (i.e. *AB_pre* and *OM_pre*) and after weight loss shown as *AB_post* and *OM_post*. Publically available data is as follows: *AB_Glut* and *AB_SC* indicate paired gluteal and abdominal subcutaneous samples, respectively, from normal weight females (GSE47513 [35]); *AB_PM* and *OM_PM* indicate data from six paired subcutaneous adipose and omentum samples, respectively, taken 12 h post-mortem from normal-weight males (GSE48472 [43]); *AB_twin* indicates subcutaneous abdominal adipose data from 642 individuals from the MuTHER twin study [46, 47] (EBI E-MTAB-1866) who had an average BMI of 26.7 (±4.9) [45]

Illumina 450K data was publically available for a number of adipose samples from lean, overweight and obese individuals. Samples accessible through MARMAL-AID [37] included those from six lean male individuals with both subcutaneous and omental adipose tissue collected at most 12 h post-mortem (GSE48472 [38]), 14 visceral adipose (of which the greater omentum is part) samples from severely obese men with (*n* = 7) and without (*n* = 7) metabolic syndrome (GSE54776 [39]), and six paired subcutaneous adipose and gluteal adipose samples from lean females (GSE47513 [30]). Illumina 450K data was also available for 642 subcutaneous adipose samples from individuals with an average BMI of 26.7 (SD 4.9) [40] from the MuTHER (Multiple Tissue Human Expression Resource) project [41, 42] (EBI E-MTAB-1866). We looked at the methylation profile of all 11 strong discriminators from the before (*n* = 10) and after (*n* = 1) weight loss data in these publically available sets and observed good agreement between the methylation profile of the probes within a tissue type irrespective of gender and/or obesity phenotype (Fig. 6).

In general, omental samples showed a tighter distribution of methylation for a given probe than did the subcutaneous adipose samples and for the majority of probes a strongly hypermethylated (mean methylation beta >0.8, cg00838040, cg17496661, cg17496661) or hypomethylated (mean methylation beta <0.2, cg09720701, cg02264990, cg22747076, cg03923561, cg22747076) phenotype. Given that the biomarker analysis was trained on data from subcutaneous and omental adipose, it is interesting to note that the samples of subcutaneous gluteal adipose tissue from lean females (‘AB_Glut’, Fig. 6) showed a methylation profile similar to that of the subcutaneous abdominal adipose samples and clearly distinct from the omental samples.

In addition, the performance of cg00838040 as a biomarker to differentiate subcutaneous adipose from omentum is clearly demonstrated in all the publically available datasets (Fig. 6 top panel), and thus, rs12102757 does not appear to impact this significantly.

## Discussion

White adipose tissue is distributed in a number of discrete depots around the body, which can be broadly split into subcutaneous (including abdominal, gluteal and femoral) and visceral (including omental, mesenteric and epiploic) [43]. Not only is the anatomical location of these adipose tissues different, but differences in their function at multiple levels (physiology, structure, cell composition, endocrinology, biochemistry, gene expression) are also observed [25, 44, 45]. Subcutaneous adipose demonstrates particular plasticity, with adipocytes, and thus tissue mass, significantly increasing in size to store excess lipid that is thought to provide buffering and protection from increased lipid accumulation [15, 16, 46–58]. By comparison, increased risk of metabolic dysfunction is associated with expansion of the omentum [21–23]. Furthermore, it is increasingly recognised that there are also differences between the different depots of one tissue type and it is likely that each depot has specific, nuanced function(s) [30, 49], with developmental genes playing a key role in this [28]. Our observations of significant differential methylation between the subcutaneous abdominal adipose and omentum are consistent with these differences between adipose tissue depots and with recently reported differences in genome-wide promoter DNA methylation between these tissues [31]. Furthermore, we observed approximately double the number of significant differentially methylated sites between the tissues after weight loss compared to beforehand. This may indicate a remodelling of adipose tissue after weight loss (and/or gastric bypass itself) to reflect a greater distinction between the tissues seen in a non-obese environment. Looking at Fig. 6 and the subcutaneous abdominal adipose samples from females before (AB_pre) and after (AB_post) weight loss in comparison to publically available subcutaneous abdominal adipose from lean females (AB_SC), 6 of 10 probes show a methylation profile for the post-weight loss sample more similar to that of the lean females, providing some support for the conjecture that the DNA methylome is reflecting a more ‘normal’ state after weight loss. Our study sampled whole adipose tissue, which is a mixture of cell types. Therefore, the methylation differences may reflect both varying complements of cell types within the different tissues investigated and the fact that cell type proportions may vary significantly within adipose tissue before and after weight loss. A possibility, reflected in our observation of almost half the number of significant differentially methylated sites before weight loss compared to after, is that the adipose tissues at this time point may well contain a greater proportion of inflammatory cells that serve to mask the difference between the tissues. In line with this suggestion, we observed a DMR unique to the before weight loss comparison within *HOTAIRM1*, which was identified as a long non-coding RNA transcribed from the region between *HOXA1* and *HOXA2* expressed specifically in cells of myeloid lineage [50]. Our previous study examining intra-adipose tissue DMRs before and after weight loss and using publically available DNA methylation data for whole blood as a proxy for inflammatory cells also highlighted this possibility [32]. Enrichment analyses of the genes mapping to the 722 DMRs observed in the tissue comparisons at both time points highlighted the role of transcriptional regulation and homeobox genes in differences between tissues. We reasoned that these are likely to represent developmental (and other) epigenetic differences that are hard-wired and critical to the function of the two tissues, because they remained consistent after the significant weight loss and other physiological and metabolic changes associated with gastric bypass. A recent study identified six adipose depot specific genes (*SORBS2*, *HAND2*, *PPARG*, *HOXC6*, *CD36* and *CLDN1*) using genome-wide promoter DNA methylation and transcriptome analysis of subcutaneous adipose and omentum from obese and lean individuals [31]. Consistent with this, we observed differentially methylated CpG loci in *SORBS2*, *HAND2*, *HOXC6* and *CLDN1*. When we looked for correlations between changes in methylation between tissues and changes in clinical parameters (for sites showing significant differential methylation at both time points), we saw strong correlations for Δbeta after weight loss and ΔHDL for CpG sites within *SORBS2* (cg04348265, *r* = 0.83), *CLDN1* (cg03623835, *r* = −0.86) and the *HOXC* cluster (*HOXC4, HOXC5, HOXC6*; cg00506343, *r* = −0.92). In addition, we observed a strong correlation between the Δbeta of four CpG sites in *ISL2* and change in HDL. Little is known about the function of *ISL2*, although the related *ISL1* gene is involved in the embryogenesis of pancreatic islet cells [51, 52] and its homology to *ISL1* implies a potential role as an enhancer of insulin gene expression [53, 54]. Given this further investigation of the role of *ISL2* in adipose tissue biology and potentially insulin sensitivity is warranted.

Not only did we see clear differences in the methylation of homeobox genes, which are known to have a role in adipose tissue development and differentiation [55], but we also observed variability in other genes with a role in development. *WT1* is involved in the mesothelial origin of visceral adipose [29], and gene expression differences between subcutaneous and visceral adipose have also been reported [56]. Consistent with this, we observed differential methylation of a number of CpG sites in the body of *WT1* (Table 3). Traditionally, DNA methylation within promoter regions has been associated with decreased transcription; however, the picture now seems considerably more complicated, with DNA methylation within the gene body affecting expression and also alternative splicing [57–59]. Therefore, given our identification of differentially methylated CpG sites within the body of *WT1* using both our analytical approaches, and this gene’s role in development and cancer biology in particular, further investigation with regard to different transcriptional isoforms or regulatory non-coding RNA transcripts may be warranted. We also note that a DMR unique to the after weight loss comparison was located in *WTAPP1* (Wilms tumour 1-associated protein pseudogene).

If significant differentially methylated loci that overlap between the two time points (before and after gastric bypass-associated weight loss) are indicative of hard-wired developmental (and functional) differences between the tissues, then we reasoned that loci ‘unique’ to each time point might provide insight(s) into the dysfunction/dysregulation that occurs in obesity and/or the subsequent effects of gastric bypass and significant weight loss.

Enrichment for gene targets of miR141 and miR200 was observed for the before weight loss comparison. These two miRNA have been reported to silence IGF2 in mouse placental development [60], and miR141 has been shown to have a role in epithelial-mesenchymal transition in kidney epithelial cells through the downregulation of *HIPK2* [61]. Oger et al. [62] observed dysregulation of miR200a and b in epididymal white adipose but not inguinal white adipose (surrogates for visceral and subcutaneous adipose in humans, respectively) in ob/ob and high-fat-diet mice. Expression was reduced in obese compared to control mice, and reduced expression was also observed in visceral, but not subcutaneous, adipose from obese type 2 diabetics compared to lean controls in humans. Furthermore, they saw changes in the relative levels of miR200a and b in preadipocytes and adipocytes in obese vs control mice, suggesting a potential role for miR200 in adipocyte differentiation. Thus, miR200 and miR141 are potentially involved in cell development and differentiation and in particular perturbations of visceral adipose associated with obesity. Our data are consistent with this and suggest a role for DNA methylation.

The homeobox gene, *HOXD8*, and the *HOX*-related long-noncoding RNA, *HOTAIRM* locus, may also warrant further investigation with regard to the dysfunction of adipose tissue in severe obesity, as DMRs in these two loci were only observed in the before weight loss comparison. As eluded above, the presence of a DMR in *HOTAIRM1* may be indicative of a greater proportion of inflammatory cells before weight loss. Other DMRs were seen at both time points within the *HOXD* cluster (*HOXD 3*, *4*, *9*, *10*). *HOXD8* lies between *HOXD3*-*4* and *HOX9*-*10*, raising the possibility that dysregulation between these two regions of the cluster is occurring.

At the after weight loss time point, DMRs in *mTOR* and *RPTOR* were observed. The mTOR signalling pathway has a fundamental role in adipose tissue function, although major questions remain regarding how this pathway is regulated in specific tissues (for a review, see [63]). Given the central role of the mTOR pathway in regulating/responding to environmental cues (such as nutrients, growth factors and stress), one would anticipate a regulatory role for epigenetics, and our data adds weight to the evidence that DNA methylation plays a part, at least, in adipose tissue.

A second aim of this study was to test the ability of our combinatorial approach to identify loci that distinguish between groups. We were motivated by (a) the hypothesis that because the two different adipose tissues had purportedly different developmental origins, they would have a clear epigenetic/DNA methylation signature and therefore present an excellent dataset to test our methodology and (b) the fact that the approach complements and extends standard statistical techniques for differential analysis. This novel method harnesses the power of mathematical abstraction via graph-theoretical techniques and gains efficiency and scalability through high-performance algorithms and implementations. Thus, unlike traditional differential analyses based on mere pairwise comparisons, a combinatorial approach interrogates the entire solution space of methylation sites and filters out all but a small subset of near-optimal discriminators (aka biomarkers).

The origins of this approach began with our work on combinatorial methods for the interpretation of gene array expression data for pulmonary adenocarcinoma [64]. While mRNA and DNA methylation data are both numeric and share many similarities, there are also some important differences; for instance the data points and thus file sizes were roughly an order of magnitude larger for the DNA methylation data. Therefore, the methods need to be both scalable (increased dimensionality) and vigilant (correction for potential false positives). While the approach concentrates mainly on extreme values, caution must be given to distributional concerns, since traditional methods very often assume normality, while probe scores on the Illumina 450K BeadChip are bimodal. Because our toolchain employs both graph-theoretical algorithms and statistical techniques, sample size is sometimes an additional concern. The addition of a dominating set filter, as described here, seems particularly effective in this regard. Moving forward, we are currently incorporating machine learning in the form of neural net classifiers into our analytical suites, but that topic is beyond the scope of the work presented here.

The utility of combinatorial approach is demonstrated by our identification of CpG sites that robustly differentiate between white adipose tissue types. These robust discriminators were filtered to reveal 10 CpG sites before weight loss and a single site after weight loss, which strongly separated the adipose tissue depots. Additional confidence in our results is provided by an excellent overlap between the pyrosequence methylation profiles of the 11 CpG sites in the discovery and validation cohorts (Figs. 3 and 4) and from publically available subcutaneous and omental adipose Illumina 450K data from lean, overweight and obese individuals (Fig. 6). With these results, we are confident that combinatorial methods are both powerful and applicable to DNA methylation data.

While there is no clinical utility in a marker to differentiate subcutaneous and omental adipose, our study provides further support for the potential of DNA methylation as a biomarker. As such, we believe future work to translate our combinatorial approach to the detection of clinically applicable DNA methylation biomarkers is warranted and may have particular merit for situations in which robust differentiators are still urgently required. Furthermore, because epigenetic mechanisms are dynamic and as such potentially reversible, these types of analyses may also highlight innovative new avenues for clinical treatment. Such analyses should take account of practicability concerns such as ease of sample/tissue collection and whether there is a biological argument for potential DNA methylation differences between sample groups. Depending on the environmental stimuli influencing the epigenetic modification, it may be that, in some cases, a tissue- and phenotype-specific change is also reflected in blood (a more tractable sample for routine analysis). For instance, a DNA methylation change within the *HIF3A* gene was recently found to be associated with BMI. On further investigation, the authors discovered the same methylation change associated with BMI in subcutaneous adipose (a phenotype-relevant tissue) but not skin [40].

It is striking that a single CpG site identified from the after weight loss comparison (cg00838040) appears to differentiate between tissue types from both lean and obese individuals (Figs. 2f, 4 and 5). This site was located in the body of *ATP2C2*, a gene that encodes a manganese-transporting calcium ATPase SPCA2 primarily located in the Golgi membrane. The CpG site (cg00838040) was located within a binding site for transcription factor CEBPB, which plays a role in adipogenesis and inflammatory responses (for a review, see [65]). A previously unreported transcript of *ATP2C2*, termed *ATP2C2c*, was recently identified in mice [66]. Transcription of this isoform is initiated in intron 23 and under epigenetic (histone modification) and transcription factor (MIST1) control. Expression of *ATP2C2* appeared to be mostly restricted to pancreatic acinar cells in mice, which plays a crucial role in the regulation of Ca^2+^ associated with the control of secretion of digestive enzymes [66]. While the intronic location of the transcription start site for *ATP2C2c* is some 30 kb away from cg00838040 and the CEBPB binding site, it raises the possibility that other, yet to be identified, isoforms of ATP2C2 exist, which may have important tissue-specific functions. Furthermore, when we looked at expression of *ATP2C2* in the GTEx Portal, levels of mRNA in subcutaneous adipose and omentum were minimal. If expression of *ATP2C2* (and/or a specific isoform) is important in adipose tissue differentiation and development, it may be that its expression occurs early in development, rather than in ‘mature’ tissue (most likely represented in the database). Thus, while a DNA methylation mark may persist, any related mRNA signature may not. While we were unable to detect any significant correlations between changes in *ATP2C2* methylation and clinical traits, our power was limited by the small sample size, and further investigation in a larger cohort is warranted.

The CpG site in *ATP2C2* interrogated by cg00838040 was effectively completely hypermethylated in all the visceral adipose tissues we examined, which almost surely reflects its discriminatory power. As such, it may be that one selection criterion that could be applied for triaging potential DNA methylation biomarkers is whether they are effectively either completely methylated or unmethylated in one group. This idea is further supported by the data for the other robust discriminators shown in Fig. 6. All but 2 of 11 probes (cg02245004 and cg09400037) show a very tight hyper- or hypomethylation profile in one of the tissues across all samples groups, cg24376776 in subcutaneous adipose and 8 probes in omentum.

While the discovery phase of our study was limited in size and gender (*n* = 15 females), 11 CpG sites which robustly separated subcutaneous and omental adipose were validated in a small, independent cohort of mixed gender (*n* = 15 before weight loss, *n* = 13/15 after weight loss, *n* = 9 males, *n* = 6 females) and in publically available datasets (*n* = 681 subcutaneous adipose and *n* = 33 omentum, mixed gender). This replication may well reflect both the power of a paired analysis and the distinctive DNA methylome signature of the two adipose tissue depots investigated. The small sample size will have significantly affected our ability to detect correlations between changes in DNA methylation between tissue and clinical parameters. As such, our observation of strong correlations (*r* > 0.7, *P* ≤ 0.001) highlights potential candidates for further investigation in larger cohorts. Our study analysed the DNA methylome of adipose tissue as a whole. However, adipose is composed of a mixed cell population that varies according to tissue type and includes adipocytes (20-40%), fibroblasts, preadipocytes, stem cells and immune cells, all of which are fundamental to its function [67]. Furthermore, DNA methylation (and other ‘global’) analyses can be confounded by cell mixtures within a sample. While there is intense work to develop algorithms to deal with such ‘admixtures’ [68–70], they have been applied to blood cell mixtures to date, and we did not have any cell type information/distribution information for the archived frozen tissue used in this study. It will be important to investigate the robust adipose tissue discriminators identified here in the different cell types within adipose tissue, for instance to determine whether cg00838040 differentiates between subcutaneous adipose and omental preadipocytes. RNA was not available for us to perform a transcriptomic analysis of the adipose tissue in parallel with the DNA methylation investigation. Given that DNA methylation is known to play a role in regulating gene expression, this highlights a potential avenue for future research, targeting some of the candidate loci identified in this study in particular.

## Conclusions

This study is, to our knowledge, the first to report a genome-wide DNA methylation comparison of subcutaneous abdominal and omental adipose before and after weight loss. We also describe the application of novel combinatorial algorithms to identify robust DNA methylation signatures that strongly differentiate between these two adipose tissue depots. Only one CpG locus in *ATP2C2* was required to distinguish between the two tissues in our study cohort, validation samples and publically available data from both lean and obese individuals. This illustrates the extreme potential power of DNA methylation as a biomarker and the overall utility of our biomarker detection approach. This work provides additional information relevant to both the development and differentiation of different adipose tissues and the potential dysfunction of these processes in obesity.

## Methods

### Sample inclusion

The cohort included individuals who underwent gastric bypass by a single surgeon at Wakefield Hospital, Wellington, New Zealand, and who returned for a second operation (incisional hernia repair (*n* = 24), incisional hernia repair and abdominoplasty (*n* = 2), silastic ring removal (*n* = 2) or Roux loop lengthening (*n* = 1)). Clinical and anthropometric data for the discovery cohort (*n* = 15 as previously described [32]) and validation cohort (*n* = 15, samples available for all 15 before weight loss and 13/15 after weight loss) are presented in Table 5. Four individuals in the discovery cohort had type 2 diabetes at the time of the first surgery (two males and two females), as did four in the validation cohort (two males and two females). Adipose tissue samples were taken at the time of surgery, immediately snap frozen in liquid nitrogen and stored at −80 °C.

### DNA extraction

DNA was extracted from approximately 100 mg of tissue using a QIAamp DNeasy Tissue Kit (Qiagen) as per the manufacturer’s protocol and included a 3-h initial lysis step and RNase treatment. Nanodrop quantitation of DNA was performed.

### DNA methylation 450K Illumina BeadChip

Analysis of Illumina 450K data from subcutaneous abdominal and omental adipose before and after gastric bypass and significant weight loss was carried out on previously generated data [32], which is available from the EBI ArrayExpress database (E-MTAB-3052).

### DNA methylation pyrosequencing

DNA samples were sent to EpigenDX (USA). Pyrosequencing assays were designed, optimised and performed by EpigenDX with both pyrograms and assay result data supplied. Loci targeted for analysis were as follows (as per Illumina 450K probe identification): cg02245004, cg03923561, cg22747076, cg09400037, cg24376776, cg11797364, cg17496661, cg09720701, cg02264990, cg01524853, cg00838040. Sufficient DNA was available from all 15 individuals in the core discovery cohort (Table 5) for samples taken before weight loss, whereas samples from only 9 of 15 were available for the after weight loss comparison.

### Biomarker analyses

We applied a combination of statistical tools and graph-theoretical algorithms to identify putative biomarkers indicative of tissue type. The general approach we employed can be traced back to our work on transcriptomic data and differential analysis toolchains [64]. Additional technical details can be found in [71, 72]. Sites are initially scored by maximizing the difference between intra-class median values, less the sum of the standard deviations within each class. A finite, simple, undirected graph is then constructed, treating individuals as vertices and weighting edges by a similarity metric using the highest scoring sites. The graph is thresholded, and cliques are isolated to check for homogeneity. If this step is successful, a parameterised implementation of red/blue dominating set is then employed to eliminate sites best covered by others [73]. Specifically, we first calculated a merit value for each methylation site, based on the site’s apparent utility in separating sample sets. We then employed graph-theoretical algorithms (primarily clique and dominating set) to identify those sites that best cover others and a Wilcoxon signed rank test to apply a *P* < 0.001 threshold. To estimate the potential effectiveness of this list, pairwise inter-sample scores were calculated using site and methylation values. If successful, homogeneous pairs should score highly, while inhomogeneous pairs should not, thus providing the desired separation.

### Data analyses

Statistical analyses relied on R [74]; combinatorial algorithms were performed in C. Quality control, normalisation and differential methylation analyses of Illumina 450K DNA methylation array data were performed as outlined previously [32]. DMRs were identified with the DMRcate package using default settings [35]. DMR overlaps were determined using GRanges intersect. All correlation analyses were performed using the base R cor function for Pearson’s correlation and the base R linear model (lm) function. PCA was performed using the FactoMineR package [75]. Publically deposited Illumina 450K data was obtained using the MARMAL-AID R package [37] or from the EBI ArrayExpress database. Gene enrichment analyses were performed using the ToppGene Suite [76]. Enrichment was classified as significant at a Bonferroni-adjusted *P* ≤ 0.05.

## Additional files

Additional file 1: Annotated data for the 3239 significantly differentially methylated CpG sites between omental and abdominal subcutaneous adipose before weight loss. Illumina probe ID, gene name, gene region and chromosomal location (as Illumina 450K manifest) are shown along with methylation statistics. OM_mean_beta and AB_mean_beta present mean beta values for omental (OM) and abdominal subcutaneous (AB) adipose, respectively. We also indicate which CpG sites overlap with those observed in the post-weight loss analysis (Overlap_CpG), whether the change in methylation between SC and OM is greater at the post-weight loss time point (Greater_Δbeta_Post) and whether the CpG site was observed as differentially methylation before vs after weight loss in AB (Overlap_AB_Benton_etal) in our previous study [37]. (No overlap with the previous OM comparison was observed). (CSV 263 kb)

Additional file 2: Annotated data for the 3239 significantly differentially methylated CpG sites between omental and abdominal subcutaneous adipose after weight loss. Illumina probe ID, gene name, gene region and chromosomal location (as Illumina 450K manifest) are shown along with methylation statistics. OM_mean_beta and AB_mean_beta present mean beta values for omental (OM) and abdominal subcutaneous (AB) adipose, respectively. We also indicate which CpG sites overlap with those observed in the post-weight loss analysis (Overlap_CpG), whether the change in methylation between SC and OM is greater at the post-weight loss time point (Greater_Δbeta_Post) and whether the CpG site was observed as differentially methylation before vs after weight loss in AB (Overlap_AB_Benton_etal) and OM (Overlap_OM_Benton_etal) in our previous study [37]. (CSV 637 kb)

Additional file 3: Annotated data for the 784 significant DMRs between omental and abdominal subcutaneous adipose before weight loss. Chromosomal location including DMR start and finish, number of CpG sites, gene symbol and level of differential methylation are included. (CSV 43 kb)

Additional file 4: Annotated data for the 1129 significant DMRs between omental and abdominal subcutaneous adipose after weight loss. Chromosomal location including DMR start and finish, number of CpG sites, gene symbol and level of differential methylation are included. (CSV 63 kb)

Additional file 5: Enrichment results for genes mapping to DMRs only observed in the before weight loss analysis. All pathways passing Bonferroni correction (*P* ≤ 0.05) are shown. (XLSX 8 kb)

Additional file 6: Enrichment results for genes mapping to DMRs only observed in the after weight loss analysis. All pathways passing Bonferroni correction (*P* ≤ 0.05) are shown. (XLSX 12 kb)

Additional file 7: Enrichment results for genes mapping to DMRs observed in both the before and after weight loss analyses. All pathways passing Bonferroni correction (*P* ≤ 0.05) are shown. (XLSX 19 kb)

Additional file 8: Enrichment results for genes mapping to DMRs observed in both the before and after weight loss analyses, for which the methylation difference between tissues was greater after weight loss. All pathways passing Bonferroni correction (*P* ≤ 0.05) are shown. (CSV 7 kb)

Additional file 9: Summary of Pearson’s correlation data passing a *P* ≤ 0.001 threshold for comparison of Δbeta (subcutaneous adipose vs omentum) and Δclinical trait (before vs after weight loss). (XLSX 19 kb)

Additional file 10: Annotated data for the 4900 CpG sites with a positive merit score in the combinatorial algorithm analysis before weight loss analysis. Illumina probe ID, gene symbol, gene region and chromosomal location (as Illumina 450K manifest) are shown along with methylation statistics. OM_mean_beta and AB_mean_beta present mean beta values for omental (OM) and abdominal subcutaneous (AB) adipose, respectively. Merit score and ranking are also provided. (CSV 310 kb)

Additional file 11: Annotated data for the 8624 CpG sites with a positive merit score in the combinatorial algorithm analysis after weight loss. Illumina probe ID, gene symbol, gene region and chromosomal location (as Illumina 450K manifest) are shown along with methylation statistics. OM_mean_beta and AB_mean_beta present mean beta values for omental (OM) and abdominal subcutaneous (AB) adipose, respectively. Merit score and ranking are also provided. (CSV 655 kb)
